# NORs on human acrocentric chromosome p-arms are active by default and can associate with nucleoli independently of rDNA

**DOI:** 10.1073/pnas.2001812117

**Published:** 2020-04-24

**Authors:** Marjolein van Sluis, Chelly van Vuuren, Hazel Mangan, Brian McStay

**Affiliations:** ^a^Centre for Chromosome Biology, School of Natural Sciences, National University of Ireland Galway, Galway H91 W2TY, Ireland

**Keywords:** acrocentric chromosomes, nucleolar organizers, nucleoli, UBF

## Abstract

Nucleoli, the sites of ribosome biogenesis and the largest structures in human nuclei, form around nucleolar organizer regions (NORs) comprising ribosomal DNA (rDNA) arrays. NORs are located on the p-arms of the five human acrocentric chromosomes. Defining the rules of engagement between these p-arms and nucleoli takes on added significance as describing the three-dimensional organization of the human genome represents a major research goal. Here we used fluorescent in situ hybridization (FISH) and immuno-FISH on metaphase chromosomes from karyotypically normal primary and hTERT-immortalized human cell lines to catalog NORs in terms of their relative rDNA content and activity status. We demonstrate that a proportion of acrocentric p-arms in cell lines and from normal human donors have no detectable rDNA. Surprisingly, we found that all NORs with detectable rDNA are active, as defined by upstream binding factor loading. We determined the nucleolar association status of all NORs during interphase, and found that nucleolar association of acrocentric p-arms can occur independently of rDNA content, suggesting that sequences elsewhere on these chromosome arms drive nucleolar association. In established cancer lines, we characterize a variety of chromosomal rearrangements involving acrocentric p-arms and observe silent, rDNA-containing NORs that are dissociated from nucleoli. In conclusion, our findings indicate that within human nuclei, positioning of all 10 acrocentric chromosomes is dictated by nucleolar association. Furthermore, these nucleolar associations are buffered against interindividual variation in the distribution of rDNA.

## Significance

A detailed description of how the genome is organized within the human nucleus is a major research goal. Nucleolar organizer regions (NORs) comprising ribosomal DNA (rDNA) arrays are located on the p-arms of the five human acrocentric chromosomes. Here we characterize the rules of engagement between NORs and nucleoli. We reveal variation in rDNA distribution, with some primary cell lines containing as many as four acrocentrics devoid of rDNA. We establish the default status of NORs as active and show that acrocentric p-arms devoid of rDNA retain nucleolar association potential. Based on our data, we propose that in diploid human cells, all 10 acrocentric chromosomes are nucleolar-associated, with involvement of non-rDNA sequences buffering against variable rDNA distribution.

Nucleoli form around arrays of ribosomal DNA (rDNA) repeats that are transcribed by the dedicated RNA polymerase I (RNA pol I) transcription machinery to produce mature ribosomal RNAs (rRNAs). In humans, rDNA arrays, the major components of nucleolar organizer regions (NORs), are located on the short arms of each of the five acrocentric chromosomes HSA13, HSA14, HSA15, HSA21, and HSA22 (1). Thus, nucleoli, the largest structures in the interphase nucleus, have the potential to provide positional cues on up to 10 of the 46 chromosomes in normal human cells. Consequently, establishing the relationship between NOR-bearing chromosomes and nucleoli is a prerequisite for describing the three-dimensional (3D) organization of the human genome in interphase nuclei (2).

Based on pulsed-field gel electrophoresis (PFGE) of intact rDNA arrays, the average rDNA content has been estimated as ∼300 repeats per genome, with rDNA arrays ranging in size from 50 kb to ∼6 Mb (3, 4). More recently, a computational approach using whole-genome DNA sequencing data has established a maximum of 410 repeats (5). Droplet digital PCR (ddPCR) is being increasingly used to measure rDNA copy number in both normal and cancer cells (6). For example, the telomerase immortalized retinal pigmented epithelial cells (hTert-RPE1) used in this study have a normal karyotype and an rDNA copy number of ∼250. Although these techniques inform us about rDNA repeat copy number, they do not provide information on their chromosomal distribution.

It has been clear for some time that from yeast to human cell lines, rDNA arrays are sufficient to induce nucleolar formation (7–9); however, this should not be interpreted as evidence that the chromosomal context of NORs plays no role (2). Studying the context of human rDNA arrays has been hampered by their omission from human genome drafts. Nevertheless, we previously assembled a distal junction (DJ) DNA sequence contig abutting rDNA arrays on their telomeric side, revealing that it is shared among the acrocentrics (10). To facilitate inclusion into genome references, we recently reported sequencing the DJ from all five acrocentrics, with ∼3 Mb of novel sequence (11). We revealed remarkable DJ sequence and functional conservation among human acrocentrics, including the production of DJ-encoded long noncoding RNAs (lncRNAs) required for nucleolar function, and also provided direct evidence of genetic exchanges between heterologous human acrocentric p-arms.

During metaphase, extensive binding by a nucleolar specific HMG-box protein, upstream binding factor (UBF), across the rDNA repeats provides a mitotic bookmark for “active” NORs (9, 12). UBF is also responsible for the two classical morphological features of active NORs on metaphase chromosomes: an undercondensed state, termed the secondary constriction, and the capability of being stained with silver nitrate (AgNORs) (13, 14). Silent NORs are not bound by UBF and thus are fully condensed. Such NORs remain transcriptionally silent in daughter cells (9, 15). At the end of mitosis, transcription of rDNA by RNA Pol I resumes. Nucleoli form around individual active NORs, which then fuse in early G1 to form mature nucleoli comprising multiple NORs (16). Mature nucleoli are associated with heterochromatin, often termed perinucleolar heterochromatin (PNH), which includes sequences surrounding rDNA arrays on acrocentric q-arms (17). Indeed, DJ sequences are located within the PNH, anchoring the linked rDNA array that projects into the nucleolar interior (10). Thus, DJs provide a unique opportunity to identify all NORs and enumerate them within mature nucleoli by fluorescent in situ hybridization (FISH).

Nucleoli have a tripartite internal organization that reflects the stages of ribosome biogenesis. Unengaged transcription factors, including UBF and RNA Pol I, are located in the fibrillar centers (FCs) (18). Transcription occurs at the interface between the FC and the dense fibrillar component (DFC) that surrounds it. Modification and early processing of nascent pre-RNAs occurs in the DFC. Released pre-rRNA species are processed and ribosome subunits assembled in the granular component (GC). Nucleoli are dynamic; induction of nucleolar stress by inhibition of RNA Pol I with actinomycin D (AMD), or by introduction of rDNA double strand breaks, results in rapid large-scale reorganization, including the formation of nucleolar caps (19). During cap formation, FC and DFC components move together with the rDNA to the nucleolar periphery (20). Caps are presumed to comprise single rDNA arrays and form adjacent to their linked DJ (10).

In this work, we address a number of major issues in relation to NORs and nucleoli. We report the distribution of rDNA among acrocentric chromosomes within nontransformed human cell lines and in human donors, revealing that a proportion of acrocentric p-arms lack detectable rDNA. We then demonstrate that the default status of NORs within nontransformed human cells is active, and that NORs associate with nucleoli regardless of their rDNA content. Finally, we show that multiple alterations in the chromosomal organization, activity status, and nucleolar association potential of NORs occur in cancer cell lines.

## Results

### rDNA Distribution Among Acrocentric p-Arms.

**Fig. 1.**
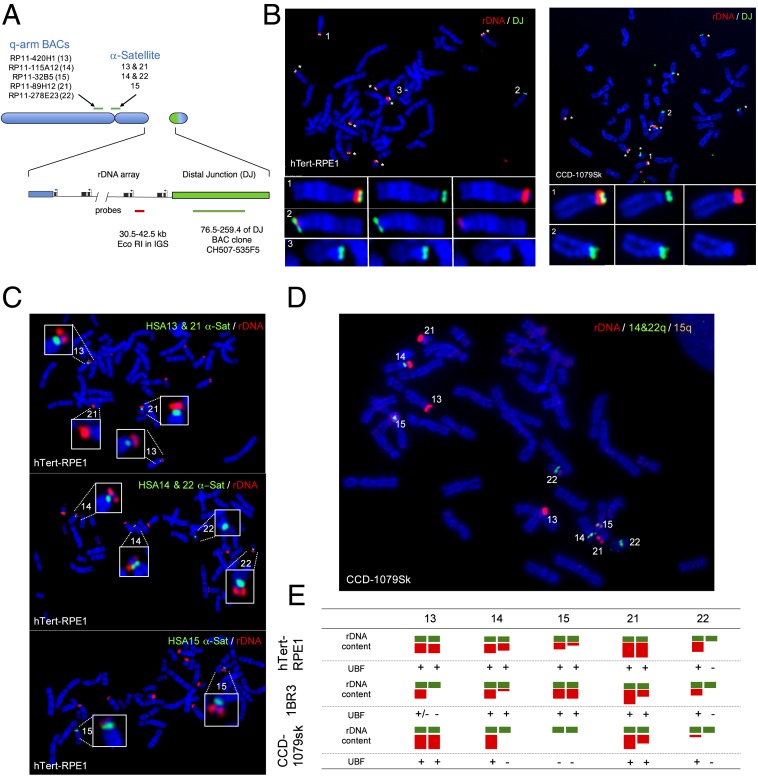
The chromosomal distribution of rDNA in nontransformed human cell lines. (*A*) Schematic showing the FISH probes used in this study. (*B*) FISH performed on metaphase spreads prepared from hTert-RPE1 (*Left*) and CCD-1079Sk (*Right*) cells. rDNA and DJ probes are in red and green, respectively, and chromosomes were stained with DAPI. (*C*) FISH performed on metaphase spreads prepared from hTert-RPE1 with rDNA (red) and α-satellite probes indicated. Note that α-satellite probes from HSA13 and HSA21 cross-react, as do HSA14 and HSA22. The HSA15 probe is unique to that chromosome. (*D*) CCD-1079Sk metaphase spreads probed with rDNA (red), HSA14 and HSA22 α-satellite (green), and HSA15 (red and green, resulting in yellow). Note that HSA13 and HSA21 are distinguished by size. (*E*) NOR ideograms for hTert-RPE1, 1BR3, and CCD-1079Sk cells. The relative rDNA contents (red) for each NOR are shown below a DJ (green). Chromosomal identities are shown on top, and UBF loading status is indicated under each NOR ideogram.

To establish the behavior of NORs in nontransformed human cells, we first determined the distribution of rDNA in hTert-RPE1 cells, as well as in 1BR3 and CCD-1079Sk primary cell lines. This was achieved using an rDNA probe from the intergenic spacer (IGS) combined with a DJ probe (Fig. 1*A*). Based on our recent DNA sequencing studies, we expected the DJ probe to hybridize with equal intensity to all NORs irrespective of their rDNA content (11). Thus, the DJ probe provides a measure of NOR genomic integrity and allows us to recognize NORs with low or no rDNA content. As expected, all acrocentric p-arms exhibited a DJ signal of uniform intensity, while the rDNA hybridization signal on acrocentric p-arms varied dramatically from high signal intensity to no detectable rDNA, as demonstrated by the enlarged individual acrocentrics in Fig. 1*B*.

Next, we combined the rDNA IGS probe with centromeric α-satellite probes to identify individual chromosomes (Fig. 1*A*). Hybridizations to hTert-RPE1 metaphase spreads revealed that one HSA22 lacked rDNA, while one HSA15 had very low levels in all observed spreads (Fig. 1*C*). With CCD-1079Sk cells, we observed four chromosomes without detectable rDNA, including both copies of HSA15 and one copy each of HSA14 and HSA22 (Fig. 1*D*). Despite this unbalanced rDNA distribution, CCD-1079Sk cells were among the first human fibroblast lines to be converted into induced pluripotent stem cells (21). In 1BR3 cells, one copy each of HSA13 and HSA22 had no detectable rDNA (*SI Appendix*, Fig. S1). These combined results are displayed as NOR ideograms in Fig. 1*E*.

GM10063 cells are mouse A9 cells containing Xder21, the product of a reciprocal translocation between HSAX and HSA21. Xder21 was previously shown to contain the most distal three rDNA repeats (22). We have also shown that it contains an intact DJ (11). By performing FISH on metaphase spreads from GM10063 mixed with either hTert-RPE1 or CCD-1079Sk, we could use the Xder21 as an internal reference for estimating rDNA content (*SI Appendix*, Fig. S2*A*). An rDNA signal on Xder21 was clearly visible, while rDNA-negative NORs were observed in both hTert-RPE1 and CCD-1079Sk spreads. Furthermore, the low-rDNA content HSA15 in hTert-RPE1 cells likely included at most a single or partial rDNA repeat.

To rule out the possibility that some NORs are populated by variant rDNA repeats with IGSs not recognized by our standard rDNA probe, we also performed metaphase FISH on spreads from CCD-1079Sk cells using a probe spanning the pre-rRNA coding sequences (*SI Appendix*, Fig. S2*B*). This probe, which included 18S and 28S rRNA coding sequences, confirmed the existence of rDNA-negative NORs.

**Fig. 2.**
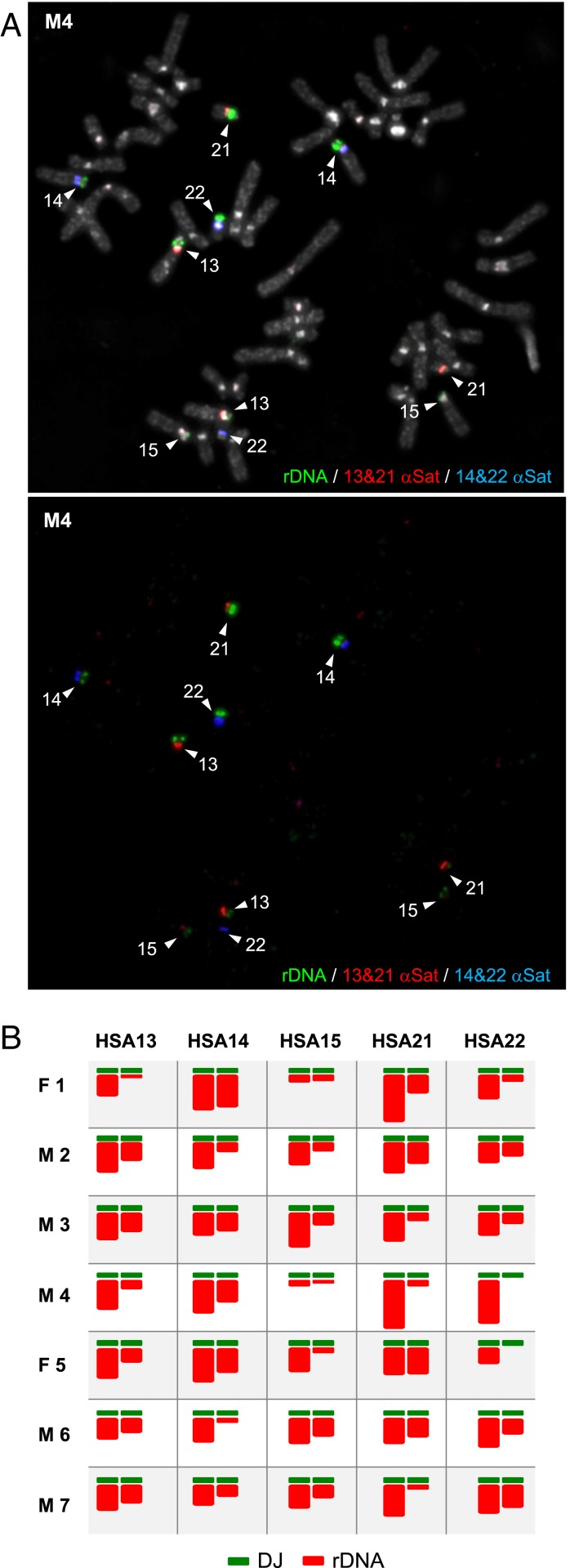
Chromosomal distribution of rDNA on metaphase spreads from human donors. (*A*) FISH performed on metaphase spreads prepared from a male donor, M4, using an rDNA (green) and α-satellite probes recognizing HSA13/21 (red) and HSA14/22 (far red, pseudocolored here in blue). Chromosomes were stained with DAPI (pseudocolored in gray). The identity of each acrocentric is indicated. (*B*) NOR ideograms showing the relative rDNA distribution in seven human donors, including M4 above (details in *Materials and Methods*).

To establish that the occurrence of acrocentric p-arms devoid of rDNA is not restricted to established cell lines, we applied the same analysis to metaphase spreads prepared from phytohemagglutinin (PHA)-stimulated peripheral blood lymphocytes of five normal male and two normal female donors. In these hybridizations, the rDNA IGS probe was combined with an α-satellite probe recognizing HSA13/21 and a second α-satellite probe recognizing HSA14/22. The advantage of combining all three probes is that we could accurately determine the relative rDNA content of each NOR while at the same time unambiguously identifying each acrocentric within single metaphase spreads. A typical hybridization from male donor 4 (M4) and NOR ideograms from all seven human donors are presented in Fig. 2. These results illustrate the widely varying distribution of rDNA among human individuals and nontransformed cell lines and identify acrocentric p-arms containing an intact DJ but apparently devoid of rDNA.

### The Default Status of NORs in Nontransformed Human Cell Lines Is Active.

**Fig. 3.**
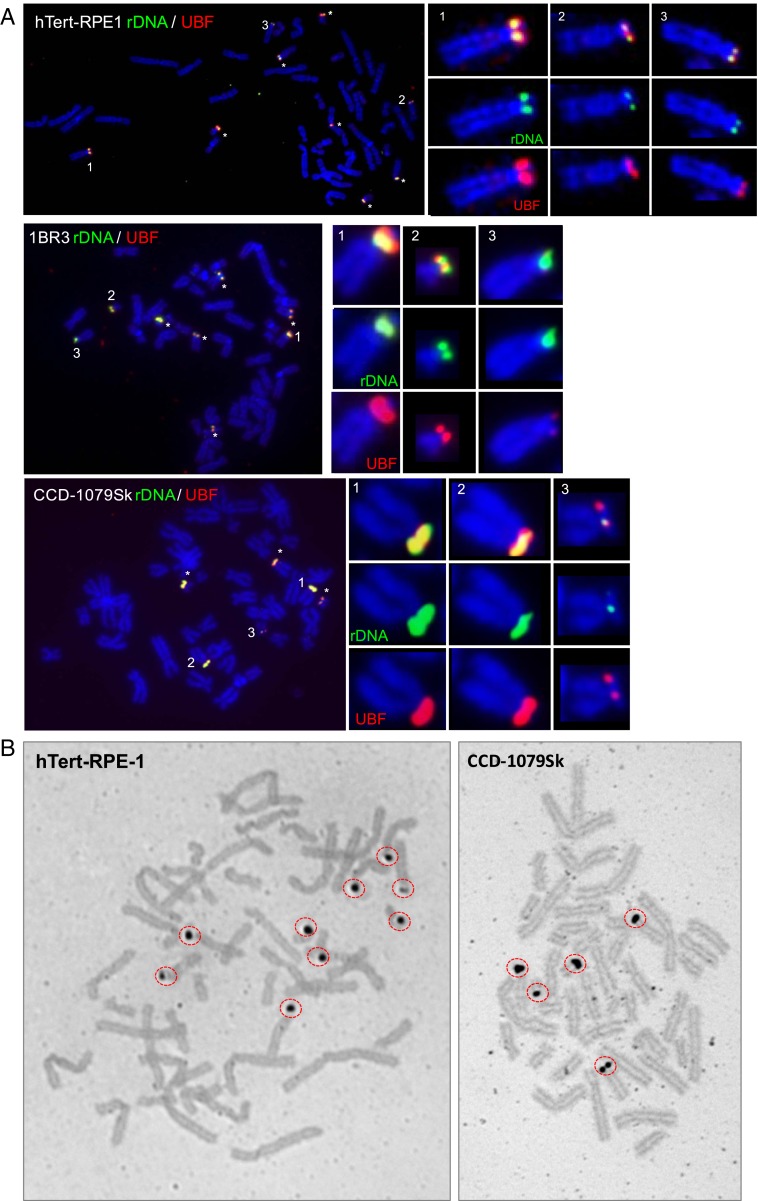
The activity status of NORs as determined by UBF loading. (*A*) Immuno-FISH performed metaphase spreads prepared from hTert-RPE1, 1BR3, and CCD-1079Sk cells visualizing rDNA (red) and UBF (green). Chromosomes were stained with DAPI. Active NORs are indicated by either an asterisk or a number. Enlarged versions of three numbered chromosomes from each cell type are shown at the right. (*B*) Silver staining performed on metaphase spreads prepared from hTert-RPE1 and CCD-1079Sk cells. Silver-positive NORs are indicated by red circles.

UBF, a mitotic bookmark for active NORs, provides a robust marker on metaphase chromosome spreads for NORs that will become transcriptionally activated in the next cell cycle (9, 12, 15). Indeed, depletion of UBF from cells results in silencing of NORs (9). Thus, we performed immuno-FISH, combining antibodies against UBF with the rDNA IGS probe, on metaphase spreads prepared from hTert-RPE1, 1BR3, and CCD-1079Sk cells (Fig. 3*A*). These results, integrated into NOR ideograms (Fig. 1*E*), show that the extent of UBF loading correlated with rDNA content. In no case did we observe an rDNA-containing acrocentric p-arm devoid of UBF; therefore, we can conclude that the default status of NORs with detectable levels of rDNA in nontransformed human cells is active.

NOR can also be visualized by silver staining of metaphase spreads, and thus are also referred to as AgNORs (23). In previous work, we showed that UBF loading is a prerequisite for silver staining of human NORs (13, 14). Having established that UBF loading on NORs positively correlates with rDNA content, we tested whether silver staining followed the same pattern (Fig. 3*B*). On metaphase spreads prepared from hTert-RPE1 cells, we routinely observed nine AgNORs, with one of them—presumably the low-rDNA HSA15—weakly positive for silver. In CCD-1079Sk spreads, we routinely observed five AgNORs. We presume that the four rDNA-negative NORs and the low-rDNA content HSA22 were negative for silver. Over the years, silver staining has been used to measure the proportion of NORs that are “active” in metaphase spreads prepared from PHA-stimulated peripheral blood lymphocytes from human donors. The observed number ranges from 7 to 10, with an average of 8 (24). With the data presented above, we can now interpret previously published silver staining patterns as a surrogate marker for the distribution of rDNA among the acrocentrics. Thus, individuals in the human population have on average two acrocentric p-arms with very few or no rDNA repeats.

### Human NORs Are Nucleolar Associated Irrespective of rDNA Content.

**Fig. 4.**
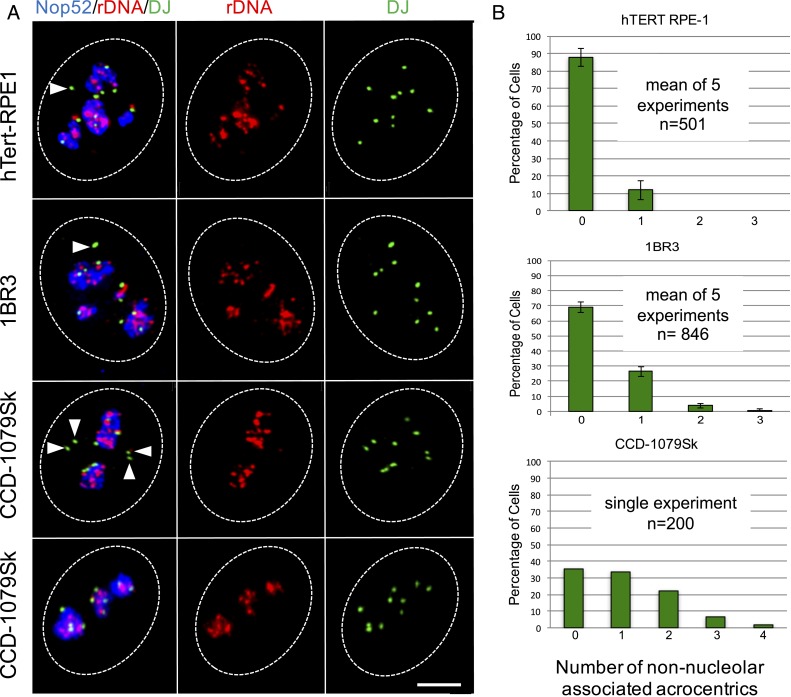
Nucleolar association of NORs. (*A*) 3D immuno-FISH performed on hTert-RPE1, 1BR3, and CCD-1079Sk cells. Nop52 antibodies (blue) used to visualize nucleoli were combined with rDNA and DJ FISH probes (red and green, respectively). Nuclear borders are indicated by dashed white lines, and NORs dissociated from nucleoli are indicated by white arrowheads. Note that to demonstrate our ability to detect nonassociated NORs, unrepresentative images for hTert-RPE1 and 1BR3 cells in which a single NOR is non–nucleolar-associated are shown. In the case of CCD-1079Sk cells, we show a cell in which four non–rDNA-containing NORs are non–nucleolar-associated and a cell in which all NORs are associated. (*B*) Quantitation of 3D immuno-FISH showing the percentage of cells in which zero, one, two, three, or four non–nucleolar-associated NORs were observed. Cell identities are shown above each bar chart. The number of experiments performed and number of cells analyzed are indicated.

We addressed the subnuclear localization of NORs within interphase cells using 3D immuno-FISH (25). Using the DJ probe, we could visualize all 10 NORs in each cell line irrespective of their rDNA content. rDNA was visualized using the IGS probe, and nucleoli were visualized using an antibody against Nop52, a GC protein involved in pre-rRNA maturation (26). Results from hTert-RPE1, 1BR3, and CCD-1079Sk cells are shown in Fig. 4. In almost 90% of hTert-RPE1 cells, all DJ signals were nucleolar-associated. In the remaining cells, such as those shown in Fig. 4*A*, a single rDNA-negative NOR was observed dissociated from nucleoli. Similarly, in most 1BR3 cells, all NORs were nucleolar-associated. In ∼25% of 1BR3 cells, a single rDNA-negative NOR was observed dissociated from nucleoli, (Fig. 4*A*). Finally, in CCD-1079Sk cells, the number of non–nucleolar-associated NORs ranged from zero (∼35% of cells) to four (∼2% of cells); Fig. 4*A* shows examples of both. As above, DJ signals that were dissociated from nucleoli were rDNA-negative.

**Fig. 5.**
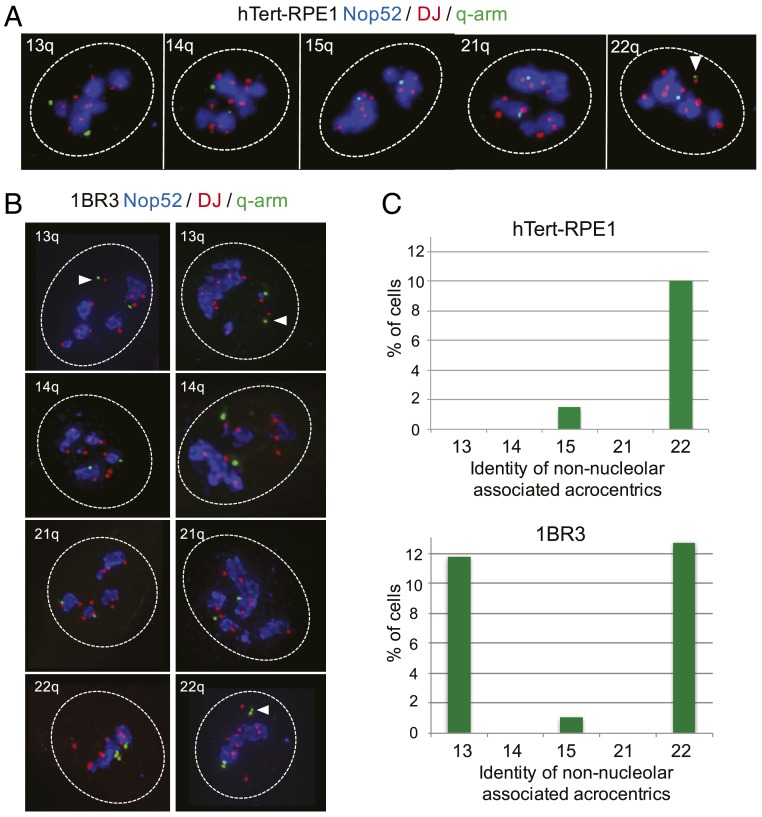
Identification of non–nucleolar-associating NORs. (*A*) 3D immuno-FISH performed on hTert-RPE1 cells. Nop52 antibodies (blue) used to visualize nucleoli were combined with DJ and q-arm BAC FISH probes (red and green, respectively). The identity of the q-arm probe is indicated in the upper right corner of each panel. Nuclear borders are indicated with dashed white lines, and the non–nucleolar-associated NOR from HSA22 is indicated by a white arrowhead. (*B*) 3D immuno-FISH performed on 1BR3 cells as above. (*C*) Quantitation of 3D immuno-FISH showing the percentage of cells in which the NOR from an identified acrocentric chromosome (shown below) is nonassociated with a nucleolus. Cell identities are shown above each bar chart. At least 100 cells were analyzed for each q-arm probe in each cell type.

We next sought to confirm the identity of NORs dissociated from nucleoli in hTert-RPE1 and 1BR3 cells using 3D immuno-FISH (Fig. 5 *A* and *B*). Bacterial artificial chromosome (BAC) clones positioned close to centromeres on each acrocentric q-arm (Fig. 1*A*) were individually combined with a DJ probe to identify NORs. In addition, Nop52 antibodies were used to visualize nucleoli (11, 25). We confirmed that in almost 90% of hTert-RPE1 cells, all NORs were nucleolar-associated. In only 10% of cells, the rDNA-negative HSA22 was dissociated, and in only ∼1.5% of cells, the low-rDNA content HSA15 was dissociated. In 1BR3 cells, we confirmed that in ∼75% of cells, all NORs were nucleolar-associated. In ∼12% of cells, the rDNA-negative HSA13 was dissociated; in ∼12% of cells, the rDNA- negative HSA22 was dissociated; and in ∼1% of cells, the low-rDNA content HSA15 was dissociated.

The results of these 3D immuno-FISH experiments allow us to draw two major conclusions. First, we can confirm that in the three nontransformed karyotypically normal cell lines that we examined, all rDNA-containing NORs were active. The second, and more surprising, conclusion is that acrocentric p-arms associated with nucleoli independent of their rDNA content.

### Genomic Rearrangements and Deregulation of NORs in Human Cancer Cell Lines.

Altered karyotypes, up-regulated ribosome biogenesis, and changes in nucleolar morphology are all associated with the transformed state in human cells (4, 27, 28). Therefore, we analyzed the chromosomal organization and activity status of NORs in three commonly used cancer cell lines: HT1080, U2OS, and HeLa.

**Fig. 6.**
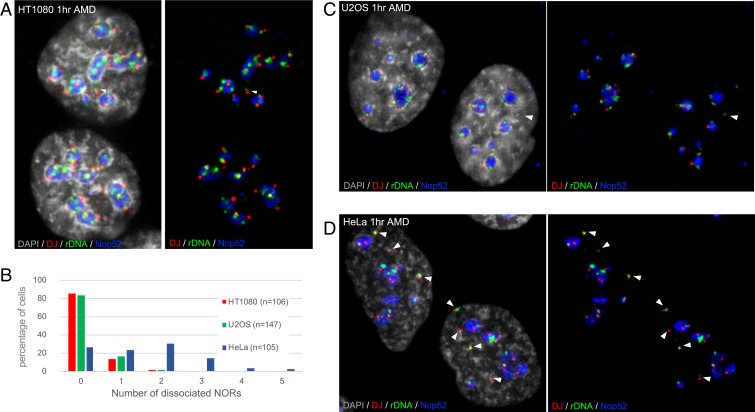
Localization of NORs in HT1080, U2OS, and HeLa cancer cell lines. (*A*) 3D immuno-FISH performed on HT1080 cells. Nop52 antibodies (far red; pseudocolored here in blue) used to visualize nucleoli were combined with DJ and rDNA FISH probes (red and green, respectively). Nuclei were visualized by DAPI staining (pseudocolored here in gray). An NOR dissociated from nucleoli in the upper cell is indicated by an arrowhead. (*B*) Quantitation of 3D immuno-FISH showing the percentages of HT1080, U2OS, and HeLa cells in which zero, one, two, three, four, or five non–nucleolar-associated NORs are observed. Cell identities and number of cells analyzed are indicated. (*C* and *D*) 3D immuno-FISH performed on U2OS cells (*C*) and HeLa cells (*D*), as above. Non–nucleolar-associated NORs are indicated by arrowheads.

HT1080 cells, a male fibrosarcoma cell line described as having a pseudodiploid karyotype, contain an activated N-ras oncogene. We first performed FISH on metaphase spreads prepared from HT1080 cells using a combination of the rDNA IGS and DJ probes (*SI Appendix*, Fig. S3*A*). The number of DJ-positive chromosomes ranged from 16 to 18. While a consistent DJ signal was observed on each acrocentric p-arm, as seen in nontransformed human cells, the rDNA signal was highly variable. We next combined α-satellite probes with the rDNA IGS probe (*SI Appendix*, Fig. S3*B*). These hybridizations confirmed that although many extra acrocentric p-arms were present in HT1080 cells, they were largely intact. The only irregularity was the presence of what appeared to be metacentric chromosomes containing an α-satellite from either HSA13 or HSA21, which were likely Robertsonian translocations (29). Finally, rDNA IGS and DJ probes were combined with Nop52 in 3D immuno-FISH on interphase cells to determine the activity status of NORs in HT1080 cells (Fig. 6*A*). In these experiments, cells were treated with AMD to induce nucleolar segregation before fixation. During nucleolar segregation, each NOR forms a distinct nucleolar cap, aiding discrimination between nucleolar-associated and -nonassociated NORs. It also facilitates NOR counting in each nucleolus (10). In ∼85% of cells, all DJ and rDNA signals were associated with nucleoli comprising multiple NORs (Fig. 6*B*). In most of the remaining cells, a single rDNA containing NOR was dissociated from nucleoli. Thus, we can conclude that, as in nontransformed human cell lines, the default status of NORs in HT1080 cells is active.

U2OS are female bone osteosarcoma epithelial cells. Metaphase spreads probed with rDNA IGS and DJ probes revealed a highly complex distribution of NOR sequences (*SI Appendix*, Fig. S4). This our first observed example of rDNA arrays being mislocated into what appears to be the q-arm of metacentric chromosomes. More tellingly, we observed uncoupling of rDNA from DJ signals. On average, nine chromosomes were positive for rDNA in each spread, four of which lacked associated DJ sequences. In one case, the rDNA had been transposed to the tip of a q-arm (*SI Appendix*, Fig. S4). Our recent work on these DJ sequences suggests that we might expect U2OS cells to exhibit unusual nucleolar morphologies (10, 11). Performance of 3D immuno-FISH on U2OS cells revealed that like HT1080 cells, all rDNA arrays (rearranged or not) appeared to be active (Fig. 6*C*). Thus in ∼82% of cells, all rDNA and DJ signals were nucleolar-associated, and in most of the remaining cells, only a single rDNA/DJ signal was nonassociated (Fig. 6*B*). Interestingly, we observed that these cells contained multiple small nucleoli derived from a single rDNA array, usually without a linked DJ. Thus, it would appear that incorporation of some chromosomes with active NORs into mature nucleoli has been impaired.

Finally, HeLa metaphase spreads probed with rDNA IGS and DJ probes identified 12 NOR-bearing chromosomes with a distribution of rDNA and DJ sequences comparable to that in nontransformed human cells (*SI Appendix*, Fig. S5*A*). DJ signals linked to rDNA signals of varying intensity were restricted to acrocentric p-arms. Hybridization with rDNA and α-satellite probes revealed a single rDNA-negative HSA13 copy. There were three copies each of HSA14 and 22 and a Robertsonian translocation involving either HSA14 or HS22 (*SI Appendix*, Fig. S5*B*). Examination of interphase cells by 3D immuno-FISH revealed the presence of silent rDNA-containing NORs in most cells (Fig. 6*D*). In >70% of the cells analyzed, at least one and up to five rDNA-containing NORs were dissociated from nucleoli (Fig. 6*B*).

To confirm that rDNA-positive NORs lacking associated Nop52 were silent, we performed 3D immuno-FISH on both untreated and AMD-treated HeLa cells using UBF antibodies (*SI Appendix*, Fig. S6). These experiments clearly identified non–nucleolar-associated rDNA-positive NORs lacking UBF, firmly establishing their silent state.

In conclusion, our detailed analysis of three cancer lines has revealed a range of NOR genotype-phenotype combinations. HT1080 cells are closest to nontransformed human cells with respect to their NOR chromosomal organization and default active status. U2OS cells also display the default active status of NORs. However, in these cells, altered NOR chromosomal organization, including loss of DJ sequences, is associated with the presence of multiple small “unfused” nucleoli. Finally, HeLa cells contain rDNA-positive NORs with apparently normal chromosomal organization that are silent and dissociated from nucleoli. We presume that such NORs are silenced by aberrant epigenetic processes, often observed in cancer cells.

## Discussion

FISH performed on metaphase spreads from nontransformed karyotypically normal human cell lines and from human donors reveals the high degree of variation in the distribution of rDNA repeats between acrocentric p-arms. Indeed, a proportion of p-arms have undetectable levels of rDNA. Based on the readily detectable human rDNA FISH signals that we observed on the Xder21 present in GM10063, the lack of detectable rDNA in human NORs can be interpreted as the presence of at most a single or partial rDNA repeat (*SI Appendix*, Fig. S2). Surprisingly, one primary cell line, CCD-1079Sk, has four such NORs, yet was among the first cell lines successfully used to generate human-induced pluripotent stem cells (21). These findings agree well with PFGE analysis of NORs showing that they can range in size from a single or partial rDNA repeat up to 6 Mb (∼140 repeats). It is likely that the loss of rDNA from acrocentric p-arms is driven by recombination. Indeed, human rDNA arrays have been described as highly recombinogenic and subject to meiotic rearrangement at a frequency of >10% per cluster per meiosis (3). Furthermore, we have recently shown that recombination events may occur between heterologous acrocentric chromosomes (11). Recombination is not limited to the rDNA array, but can also occur distal to the rDNA. Finally, recombination within rDNA arrays might not be restricted to meiosis. However, we should point out that the rDNA distribution is fixed within normal cell lines and the donor metaphase spreads that we have examined.

Alterations in the chromosomal arrangement of NORs are observed in cancer lines. Most dramatic are those observed in U2OS cells, where rDNA and DJ sequences have become uncoupled and rDNA arrays can be found in the middle of chromosomes. We speculate that DJ-negative rDNA arrays yield nucleoli that remain isolated and unfused with other NORs/nucleoli. Interestingly, U2OS cells are deficient in ATRX (alpha thalassemia/mental retardation X-linked). Moreover, rDNA copy loss and repeat instability are features of ATRX-mutated cancers (30).

It is commonly stated that the number of rDNA repeats present in the genome of most organisms is surplus to requirements and that within most cells, ∼50% of repeats are epigenetically silenced (reviewed in ref. 31). Thus, at the outset of this work, we imagined that a proportion of NORs in human cell lines would be silent. Indeed, early observations from HeLa cells supported this view (15, 32). Therefore, it was surprising to find that all rDNA-containing NORs in nontransformed human cell lines were “active,” as defined by loading of the mitotic bookmarker protein UBF. We confirmed that silent rDNA-containing NORs are present in HeLa cells but rarely seen in two other cancer lines, HT1080 and U2OS. Currently, there are insufficient data to determine the proportion of rDNA repeats that are silent in nontransformed human cells, including those examined here. Interestingly, methylation of rDNA promoter regions, considered a marker for silent repeats, is not observed in human peripheral human blood DNA (33). Whatever their proportion, we presume that silent repeats are interspersed with active repeats. In contrast to our results in human cells, research in plants such as *Arabidopsis* has revealed very clear evidence for silencing of entire NORs, involving both “active” epigenetic processes and chromosomal context (34).

Our second surprising finding is that in normal human cell lines, NORs are nucleolar-associated irrespective of their rDNA content. For example, the p-arm of a single HSA22 in hTert-RPE1 cells has at most one rDNA repeat, yet is nucleolar-associated in ∼90% of cells. Likewise, in CCD-1079Sk, the four apparently rDNA- negative NORs are nucleolar-associated in the vast majority of cells. These findings suggest that either a single rDNA repeat is sufficient to drive nucleolar association of acrocentric chromosomes or other sequences on the p-arm drive nucleolar association. We have previously shown that synthetic arrays of DJ sequences integrated into metacentric human chromosomes are nucleolar-associated (10). We have also demonstrated that human acrocentric chromosomes present within mouse monochromosomal somatic cell hybrids are nucleolar-associated even though human rDNA arrays are transcriptionally silent in this context (11, 35). Considering these findings, it seems highly likely that the association of multiple acrocentric p-arms into a large mature nucleolus is driven by the activity or properties of non-rDNA sequences. At present, it is unclear why in a small proportion of cells, rDNA-negative NORs are dissociated from nucleoli. The possibility that this reflects either a particular cell cycle stage or withdrawal from the cell cycle is an area for future investigation.

In recent years, liquid-like properties have been used to explain key aspects of nucleolar organization (20). Of particular relevance here, experiments with amplified nucleoli in *Xenopus* germinal vesicles revealed a liquid droplet-like behavior that can facilitate nucleolar fusion (36). Critically, such amplified nucleoli form around episomal rDNA repeats, untethered to chromosomes. Our data reveal that acrocentric p-arms devoid of rDNA and nucleolar material are gathered into mature nucleoli in human cells. Thus, we conclude that generation of such mature nucleoli comprising multiple NORs is not driven primarily by the liquid-like behavior of nucleoli. As it currently stands, we do not know the mechanisms involved in this generation, but it is likely related to the mechanism whereby nucleolar-associated domains on nonacrocentric chromosomes and the inactive X chromosome in female cells associate with nucleoli (17, 37, 38).

In conclusion, we have now established that the default status of NORs in nontransformed human cells is active and nucleolar-associated. Our findings have direct consequences for genome organization within the human nucleus; specifically, all 10 acrocentric chromosomes are likely associated with nucleoli. Our observation that nucleolar association of acrocentric-p-arms is independent of rDNA content is of particular relevance, as this would provide a buffer against the varying distribution of rDNA among individuals and cell lines that we have observed. Our findings with CCD-1079Sk cells illustrate this point. These cells have four acrocentric p-arms without detectable rDNA and one acrocentric p-arm with at most a few repeats. Despite this unusual rDNA distribution, these cells can be efficiently converted into iPS cells that maintain the developmental potential to differentiate into advanced derivatives of all three primary germ layers (21). Finally, in transformed cells, in which large-scale genome instability has been observed (39), we can now report that this includes alterations in the chromosomal organization of NORs, their default active status, and their nucleolar-association potential. This would be expected to have significant knock-on consequences for genome organization and expression throughout the nucleus.

## Materials and Methods

### Cell Lines.

Karyotypically normal hTERT RPE-1 cells (ATCC CRL-4000) were derived by transfecting the retinal pigmented epithelial line RPE-340 cell line with pGRN145 hTERT-expressing plasmid (40). Cells were maintained in DMEM/F-12 Ham (1/1) medium (Sigma-Aldrich) containing 2 mM l-glutamine, 10% (vol/vol) FBS, and 0.25% (vol/vol) sodium bicarbonate. Human male newborn skin fibroblasts, CCD-1079Sk (ATCC CRL-2097), are karyotypically normal. CCD-1079Sk fibroblasts and the normal adult male fibroblast line 1BR3 were maintained in DMEM/F-12 Ham (1/1) medium containing 2 mM l-glutamine, 10% (vol/vol) FBS, and nonessential amino acids. Cancer cell lines HT1080 and HeLa were cultured in DMEM (Gibco) with 10% FBS. U2OS cells were grown in McCoys 5A (Sigma-Aldrich) supplemented with 10% FBS. GM10063, A9 cells containing an Xder21 reciprocal translocation product, were obtained from the Coriell Institute and cultured as specified. Cell line authentication was done by short tandem repeat profiling (Eurofins).

### Probes Used in FISH.

We used the plasmid pUC-hrDNA-12.0 to detect human rDNA. This plasmid contains a 12.0-kb EcoRI fragment that corresponds to sequences between 30.5 and 42.5 kb of the human rDNA repeat (GenBank accession no. U13369). rDNA (18S/28S) probes comprised two EcoRI fragments encompassing the pre-rRNA coding sequences of the rDNA repeat. We visualized DJ sequences using BAC clone CH507-535F5 (GenBank accession no. CT476834). BAC clones from acrocentric q-arms positioned near centromeres were identified by consulting the human BAC resource (https://www.ncbi.nlm.nih.gov/genome/cyto/hbrc.shtml). Clones RP11-420H1 (GenBank accession no. AC018739), RP11-115A12 (GenBank accession no. AC010766), RP11-32B5 (GenBank accession no. AC068446), RPCI-11-89H21 (GenBank accession no. AZ517782), and RP11-278E23 (GenBank accession no. AC013360), were selected as probes for HSA13, HSA14, HSA15, HSA21, and HSA22, respectively. All BACs were obtained from the BACPAC Resource Center, Children's Hospital Oakland Research Institute. The α-satellite probes were prepared as described previously (11). All FISH probes were directly labeled by nick-translation using Green 496 dUTP, Red 580 dUTP, or Far Red 650 dUTP (Enzo).

### Antibodies.

Antibodies against human UBF and Nop52, raised in sheep against full-length recombinant proteins produced in insect cells and bacteria, respectively, have been described elsewhere (13, 41). All secondary antibodies were obtained from Jackson ImmunoResearch.

### Metaphase FISH.

Cells in T75 flasks were grown to ∼70% confluence and then treated for 30 min to 1 h with demecolcine (Sigma-Aldrich) at a final concentration of 0.1 μg/mL. Cells were harvested by trypsinization and pelleted by centrifugation. The cell pellet was gently resuspended in 3 to 5 mL of 75 mM KCl and incubated for 30 min at 37 °C. A few drops of cold fixative (3:1 [vol/vol] methanol:acetic acid prepared freshly and stored at −20 °C) were added to the cells, followed by spinning at 1,000 rpm for 5 min. The cell pellet was resuspended in 5 mL of cold fixative, added dropwise while flicking the tube to mix. The cells were centrifuged at 1,200 rpm for 5 min at 4 °C and then resuspended in an appropriate volume of fixative and stored at −20 °C. Cells were dropped onto microscope slides and aged for at least 24 h at room temperature, followed by denaturation and hybridization as described previously (10, 11). Normal human metaphase slides from seven donors (5 male and 2 female) were obtained from Applied Genetics Laboratories.

### Immuno-FISH of Metaphase Spreads.

Metaphase spreads were prepared as described above and used immediately without aging. Slides were first fixed with 1% paraformaldehyde (wt/vol) in phosphate buffered saline (PBS) for 10 min and then subjected to FISH as above. After the posthybridization washes, slides were washed with PBS. Primary UBF antibody, diluted in 4% BSA (wt/vol) in PBS, was applied, and the slides were incubated at 37 °C overnight. Following three 10-min phosphate buffered saline (PBS) washes, slides were incubated with secondary antibody for 2 h at 37 °C. After another three 10-min washes with PBS, slides were mounted with Vectashield plus DAPI.

### 3D Immuno-FISH on Interphase Cells.

For 3D immuno-FISH, cells grown on Superfrost Plus microscope slides (VWR) were fixed, denatured, probed, and antibody-stained as described previously (10, 25). Nop52 antibody staining was performed after FISH.

### Microscopy.

Images were captured and processed as described previously (11). The rDNA content of individual acrocentrics from seven donors (five males and two females) was determined using the quantification module in LASX 3.3 software (Leica). For each individual, seven spreads were quantified. Chromosome identities were determined using specific α-satellite probes as described previously (11). For each donor, the mean corrected rDNA hybridization signal was determined by subtracting background signal from equivalent volumes on acrocentric q-arms. The rDNA content of each acrocentric was then expressed as a percentage of the total rDNA hybridization signal.

### Data Availability.

All data are presented in the main texts or are available in *SI Appendix*. The plasmid constructs used in this study are available from the corresponding author.

## Supplementary Material

Supplementary File
