# Supplementary material for: NORs on human acrocentric chromosome p-arms are active by default and can associate with nucleoli independently of rDNA
Source: Proc Natl Acad Sci U S A. 2020 Apr 24;117(19):10368–77. doi: 10.1073/pnas.2001812117 (PMC7229746; doi:10.1073/pnas.2001812117)
Supplement: Supplementary File [file pnas.2001812117.sapp.pdf]

PNAS
www.pnas.org

Supplementary Information for

NORs on human acrocentric chromosome p-arms are active by default and can associate with nucleoli independently of rDNA

Marjolein van Sluis[1], Chelly van Vuuren[1] Hazel Mangan and Brian McStay*.

***Corresponding author:** Brian McStay.

Email: brian.mcstay@nuigalway.ie

This PDF file includes:

Figures S1 to S6

www.pnas.org/cgi/doi/10.1073/pnas.2001812117

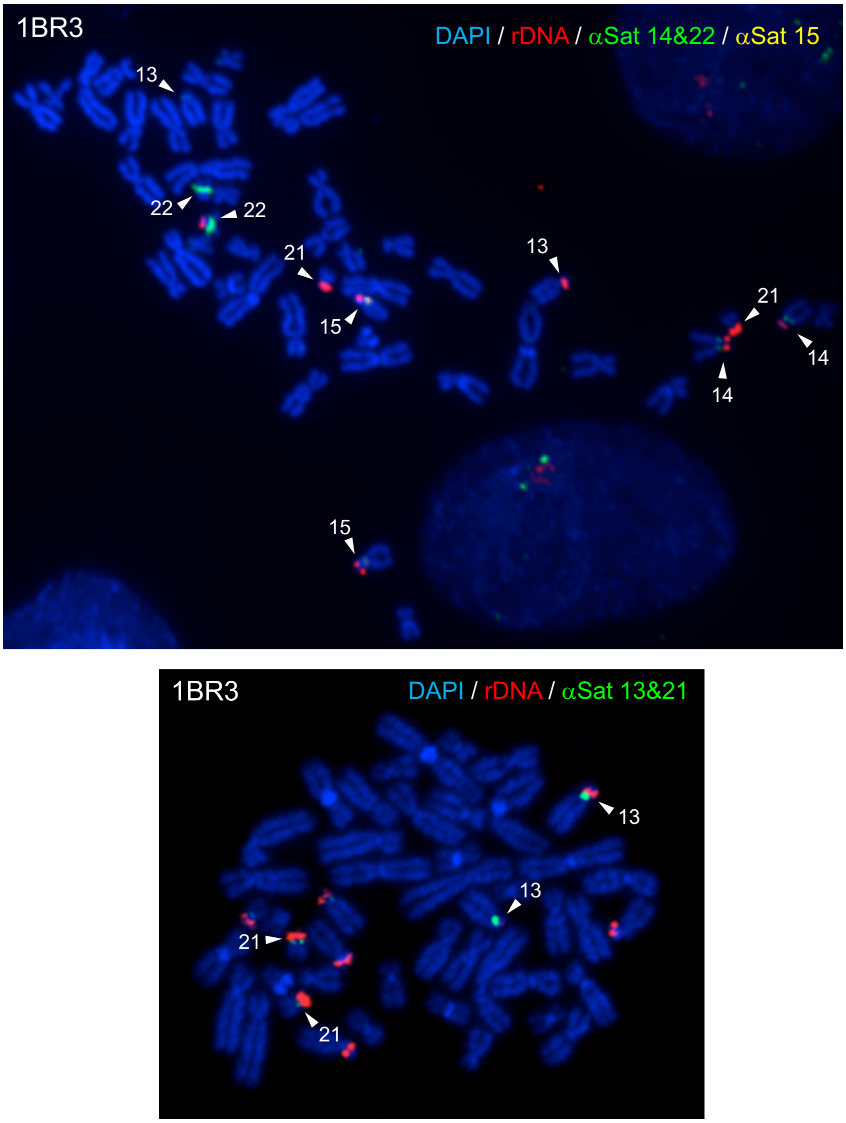

Fig. S1. The chromosomal distribution of rDNA in 1BR3 cells. FISH performed on metaphase spreads prepared from 1BR3 cells using an rDNA (red) and α-satellite probes. In the top panel, α-satellite probes recognizing HSA14/ 22 (green) and HSA15 (yellow, a mixture of red and green) were used. In the bottom panel, an a-satellite probe recognizing HSA13/ 21 (green) was used. Chromosomes were DAPI stained. The identity of each acrocentric is indicated.

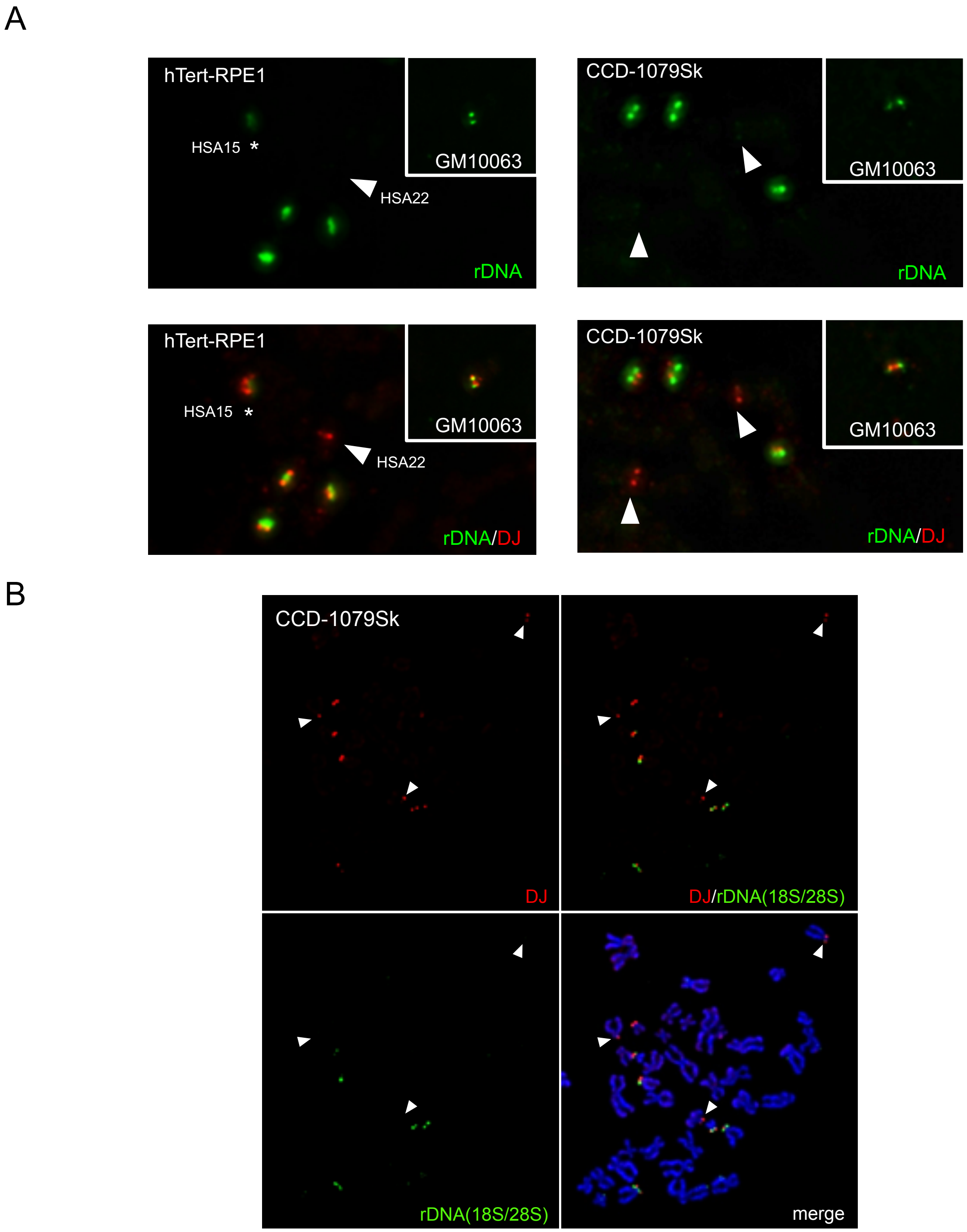

Fig. S2. Acrocentric p-arms devoid of rDNA. (A) Mixed metaphase spreads from hTert-RPE1 and GM10063 cells (left panels) or CCD-1079Sk and GM10063 cells (right panels) were probed with rDNA and DJ probes. Sections from a typical hTert-RPE1 or CCD-1079Sk spread are shown together with the Xder21 hybridization signal from an adjacent GM10063 spread (insets). rDNA negative NORs are indicated by arrowheads and the low rDNA HSA15 in hTert-RPE1 by an asterisk. Note that all spreads were captured using the same exposure settings and figures were prepared using identical image enhancements. (B) CCD-1079Sk chromosome spreads were probed using rDNA (18S/28S) and DJ probes combined. rDNA negative NORs are indicated by arrowheads .

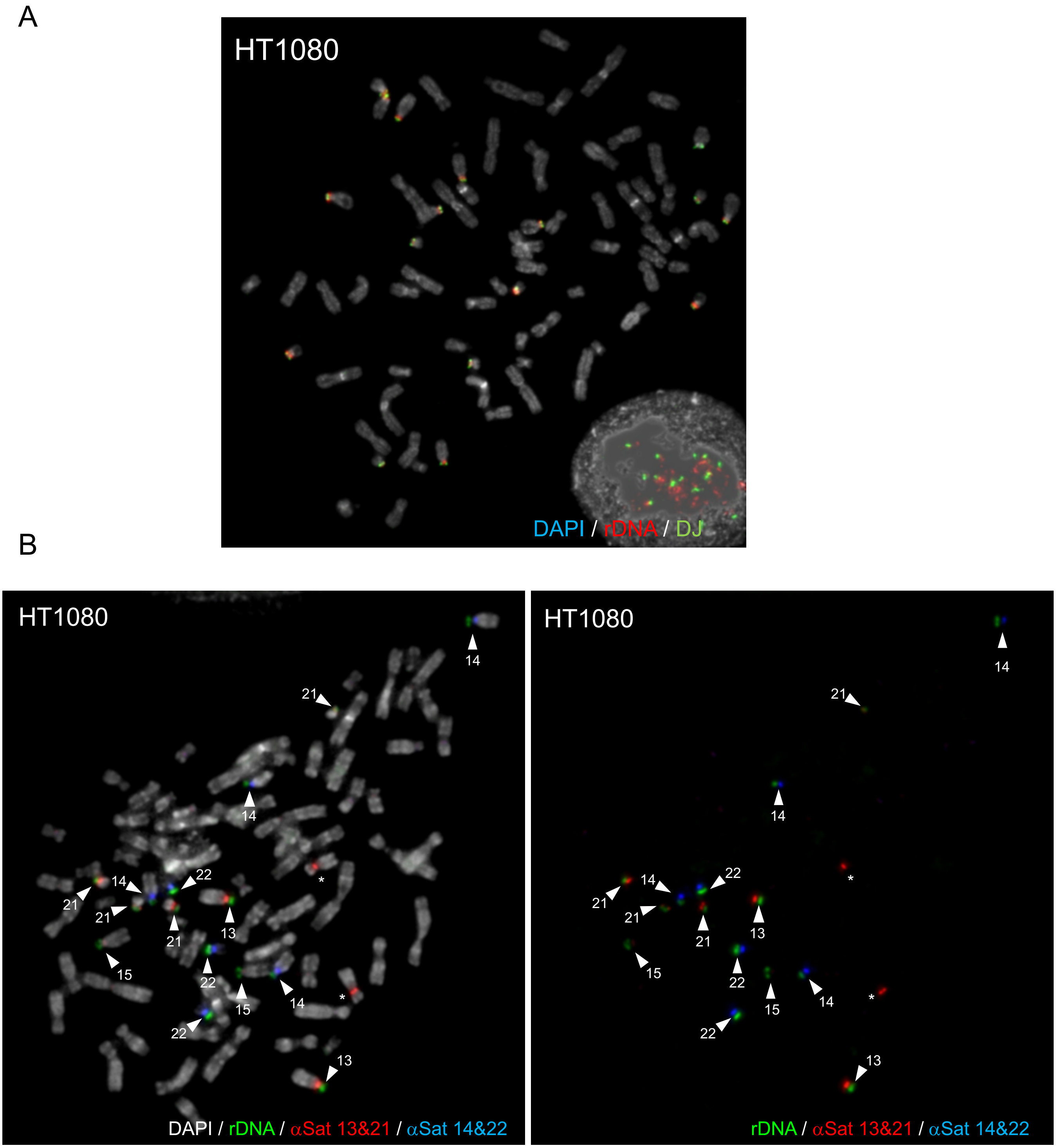

Fig. S3. The chromosomal organization of rDNA on metaphase spreads from HT1080 cells. (A) FISH was performed on metaphase spreads with rDNA (red) and DJ (green) probes. (B) Spreads were probed with rDNA (green), α-satellite probe recognizing HSA13/21 (red) and α-satellite probe recognizing HSA14/22 (far red, pseudo-colored in blue). Chromosomes were DAPI stained (pseudo-colored in grey). The identity of chromosomes are indicated. Robertsonian translocations involving either HSA13 or HS21 are indicated by an asterisk.

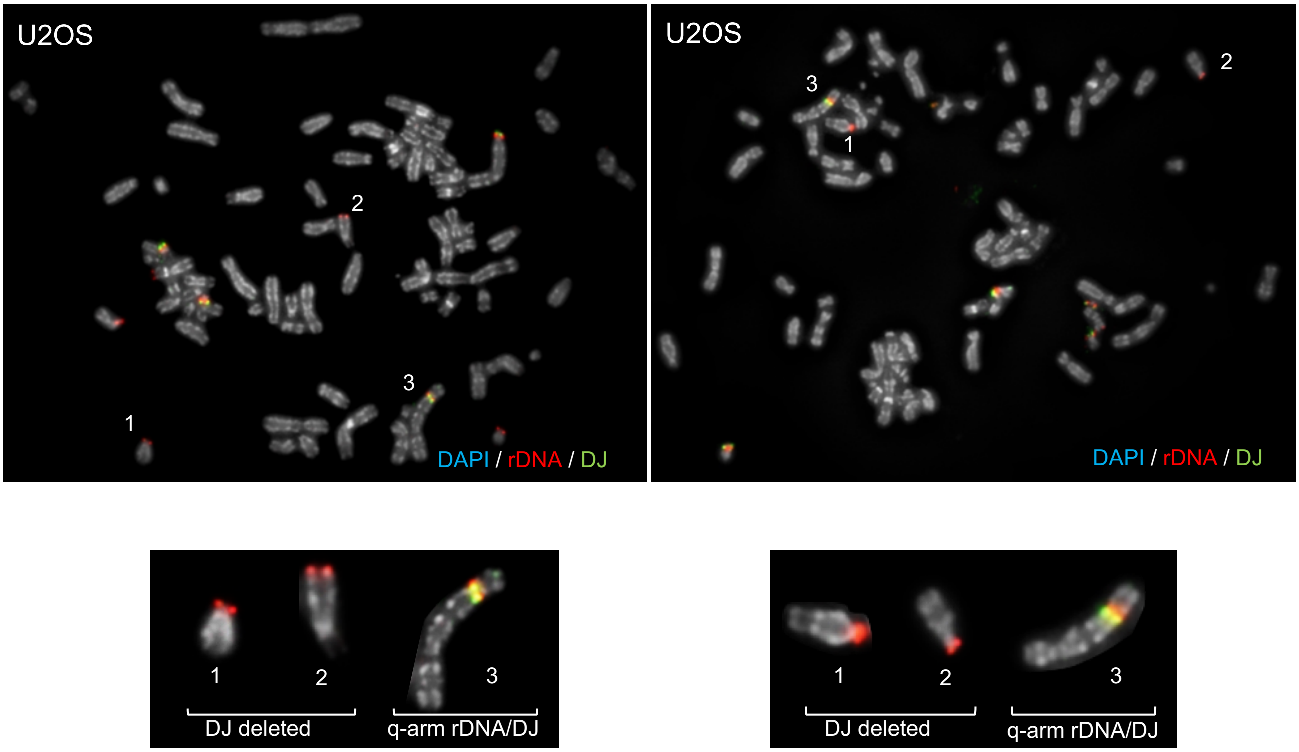

Fig. S4. The chromosomal organization of rDNA on metaphase spreads from U2OS cells. FISH was performed on metaphase spreads with rDNA (red) and DJ (green) probes. Chromosomes were DAPI stained (pseudo-colored in grey). Enlarged versions of indicated single chromosomes are shown below. These include DJ deleted chromosomes and chromosomes with apparent q-arm rDNA/DJ signals.

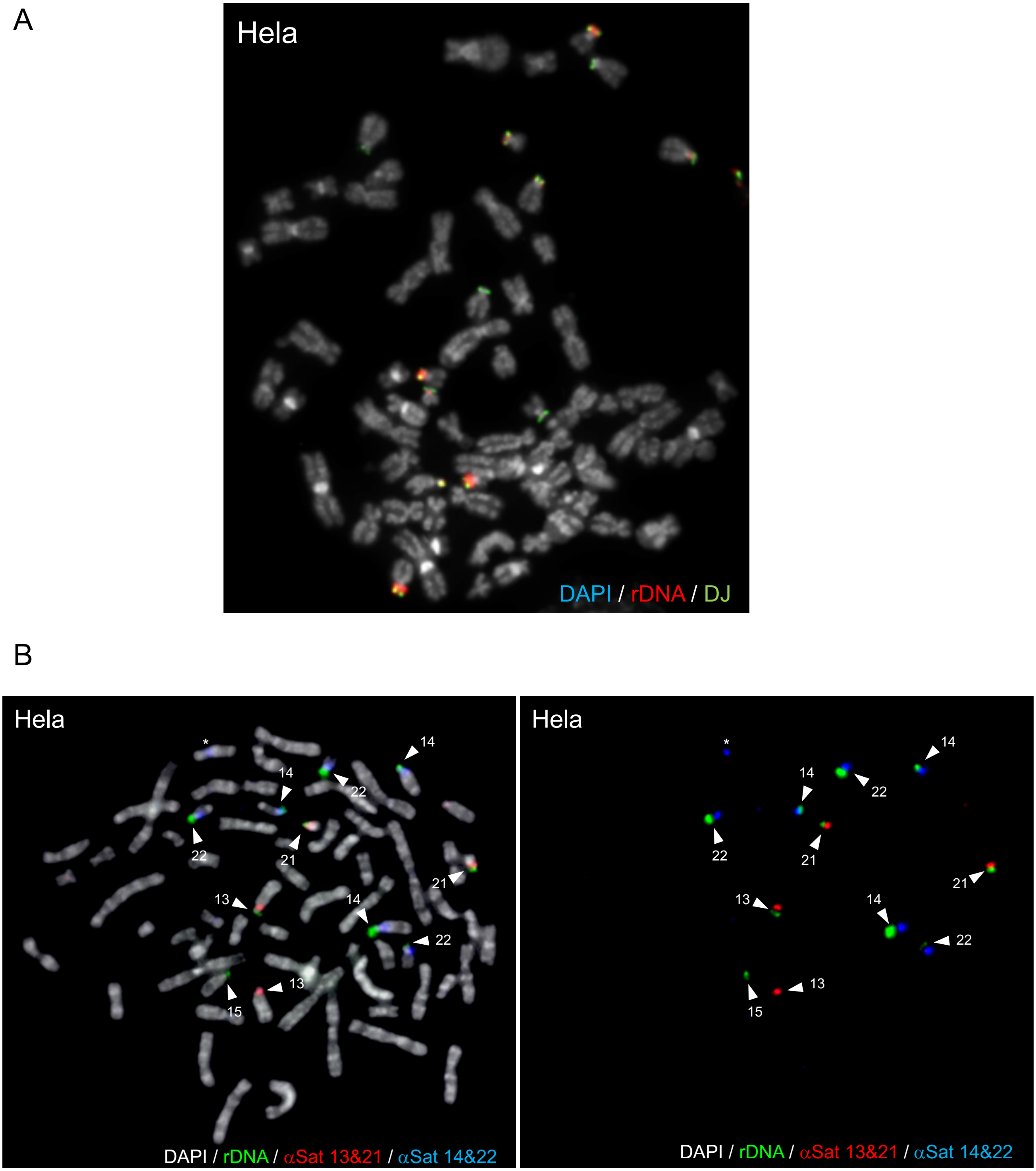

Fig. S5. The chromosomal organization of rDNA on metaphase spreads from HeLa cells. (A) FISH was performed on metaphase spreads with rDNA (red) and DJ (green) probes. (B) Spreads were probed with rDNA (green), α-satellite probe recognizing HSA13/21 (red) and α-satellite probe recognizing HSA14/22 (far red, pseudo-colored in blue). Chromosomes were DAPI stained (pseudo-colored in grey). A Robertsonian translocation involving either HSA14 or HS22 is indicated by an asterisk.

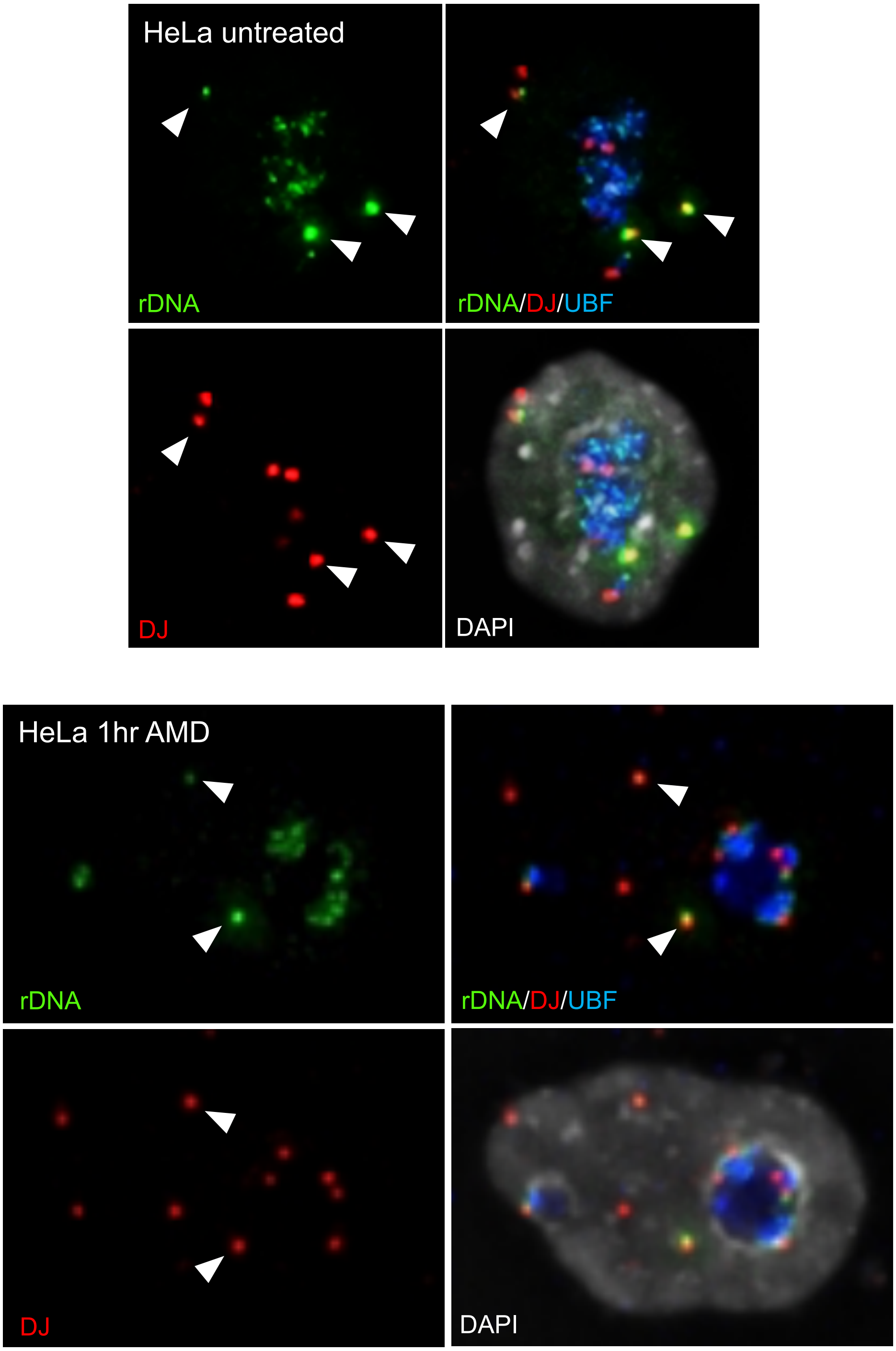

Fig. S6. Silent rDNA containing NORs in HeLa cells are devoid of UBF. 3D-immunoFISH was performed on untreated and AMD treated HeLa cells (upper and lower panels respectively) using rDNA and DJ probes combined with hUBF antibodies. Silent (non-UBF associated) rDNA containing NORs are indicated by arrowheads
